# DNA methylation and histone deacetylation associated with silencing DAP kinase gene expression in colorectal and gastric cancers

**DOI:** 10.1038/sj.bjc.6600319

**Published:** 2002-06-05

**Authors:** A Satoh, M Toyota, F Itoh, T Kikuchi, T Obata, Y Sasaki, H Suzuki, A Yawata, M Kusano, M Fujita, M Hosokawa, K Yanagihara, T Tokino, K Imai

**Affiliations:** First Department of Internal Medicine, Sapporo Medical University, S-1, W-16, Chuo-ku, Sapporo 060-8543, Japan; Department of Molecular Biology, Cancer Research Institute, Sapporo Medical University, Sapporo 060-8556, Japan; Keiyukai Sapporo Hospital, Sapporo 003-0027, Japan; National Cancer Center Research Institute, Tokyo 104-0045, Japan

**Keywords:** DNA methylation, histone acetylation, chromatin

## Abstract

Death-associated protein kinase is a positive regulator of programmed cell death induced by interferon γ. To investigate the role of epigenetic inactivation of death-associated protein kinase in gastrointestinal cancer, we examined the methylation status of the 5′ CpG island of the death-associated protein kinase gene. Methylation of the 5′ CpG island was detected in 3 of 9 colorectal and 3 of 17 gastric cancer cell lines, while among primary tumours, it was detected in 4 of 28 (14%) colorectal and 4 of 27 (15%) gastric cancers. By contrast, methylation of the edge of the CpG island was detected in virtually every sample examined. Death-associated protein kinase expression was diminished in four cell lines that showed dense methylation of the 5′ CpG island, and treatment with 5-aza-2′-deoxycitidine, a methyltransferase inhibitor, restored gene expression. Acetylation of histones H3 and H4 in the 5′ region of the gene was assessed by chromatin immunoprecipitation and was found to correlate directly with gene expression and inversely with DNA methylation. Thus, aberrant DNA methylation and histone deacetylation of the 5′ CpG island, but not the edge of the CpG island, appears to play a key role in silencing death-associated protein kinase expression in gastrointestinal malignancies.

*British Journal of Cancer* (2002) **86**, 1817–1823. doi:10.1038/sj.bjc.6600319 www.bjcancer.com

© 2002 Cancer Research UK

## 

Death-associated protein kinase (DAPK) was initially identified as a positive mediator of programmed cell death induced by interferon γ (Deiss *et al*, 1995), though it is now known to also be involved in cell death induced by FAS, tumour necrosis factor α, and detachment from extracellular matrix (Inbal *et al*, 1997; Cohen *et al*, 1997, 1999). DAPK also suppresses oncogene-induced transformation induced by p19ARF-dependent activation of p53 (Raveh *et al*, 2001), thus regulating a key apoptotic checkpoint in human tumours. As disruption of processes involved in programmed cell death is a common feature of human cancer, it is significant that DAPK was recently shown to be inactivated by promoter methylation in a variety of human tumours, including B cell lymphoma, small cell lung cancer and multiple myeloma (Kissil *et al*, 1997; Katzenellenbogen *et al*, 1999; Esteller, 2000; Tang *et al*, 2000; Dong *et al*, 2001; Kim *et al*, 2001; Ng *et al*, 2001).

There is now compelling evidence that DNA methylation plays a key role in silencing gene expression during the progression of gastrointestinal cancer (Jones and Laird, 1999; Toyota and Issa, 1999; Baylin *et al*, 2001). We previously showed that p16INK4A, E-cadherin, hMLH1 and 14-3-3sigma are all inactivated in gastric cancers by promoter hypermethylation (Suzuki *et al*, 1999, 2000), and that methylation of p16INK4A and hMLH1 is frequently detected in colorectal and gastric cancers associated with the CpG island (CGI) methylator phenotype (Toyota *et al*, 1999a,b). The precise molecular mechanism responsible for DNA methylation-dependent gene silencing remains unclear; however, recent findings suggest the involvement of histone deacetylation (Bird and Wolffe, 1999; Magdinier and Wolffe, 2001; Nguyen *et al*, 2001). Indeed, inhibition of histone deacetylation acts synergistically with inhibition of DNA methylation (Cameron *et al*, 1999) to induce gene expression.

To clarify the molecular mechanism involved in silencing DAPK expression in gastrointestinal cancer, we examined the DNA methylation and histone acetylation status of the 5′ CGI of the DAPK gene in a panel of cell lines and primary cancers. Our results indicate that aberrant methylation and histone deacetylation of the region around the transcription start site is closely associated with the loss of DAPK expression, and that methyltransferase and histone deacetylase inhibitors act synergistically to restore expression. The use of such drugs may thus represent an effective new approach to the treatment of colorectal and gastric cancer.

## MATERIALS AND METHODS

### Cell line and tissue

Nine colorectal and 17 gastric cancer cell lines were used for methylation analysis. Of these, all of the colorectal and nine of the gastric cancer cell lines were obtained either from the American Type Culture Collection (Manassas, VA, USA) or from the Japanese Collection of Research Bioresources (Tokyo, Japan). The remaining eight gastric cancer cell lines (HSC39, HSC40, HSC41, HSC42, HSC43, HSC44, HSC45 and SH-101) were established by K Yanagihara (Yanagihara *et al*, 1991, 1993). In addition, 28 primary colorectal and 27 gastric cancer specimens as well as two specimens of normal colon mucosa and two of normal stomach mucosa were obtained from the Department of Surgery, Sapporo Keiyukai Hospital (Suzuki *et al*, 2000).

All cell lines were cultured in appropriate medium. DNA was extracted using the phenol/chloroform extraction method, while total RNA was extracted using ISOGEN (Nippon Gene, Japan) according to the manufacture's instructions. To analyse restoration of DAPK expression, MKN28 and HSC44 cells were incubated for 96 h with 1 μM 5-aza-2′-deoxycytidine (5-aza-dC) (SIGMA, St. Louis, MO, USA) and/or for 24 h with 300 nM trichostatin (TSA), a histone deacetylase inhibitor (WAKO, Tokyo, Japan), after which they were harvested and their RNA extracted for further analysis.

### Combined bisulphite restriction analysis (COBRA)

Initially, genomic DNA was treated with sodium bisulphite (SIGMA) as described previously (Clark *et al*, 1994). Briefly, 2 μg of DNA were denatured for 10 min at 37°C in 2 M NaOH, after which 30 μl of 10 mM hydroquinone (Sigma Chemical Co) and 520 μl of 3 M sodium bisulphite (pH 5.0) were added, and the resultant mixture was incubated for 16 h at 50°C. The modified DNA was then purified using a Wizard DNA Purification System (Promaga, Madison, WI, USA), after which it was again treated with NaOH and precipitated. Finally, the DNA precipitate was resuspended in 20 μl of TE buffer and stored at −20°C until use.

Combined bisulphite restriction analysis (COBRA), a semi-quantitative methylation analysis, was carried out as described previously (Xiong and Laird, 1997). PCR was performed in a volume of 50 μl containing 1×PCR buffer (67 mM Tris-HCl, pH 8.8, 16.6 mM (NH_4_)_2_SO_4_, 6.7 mM MgCl_2_, and 10 mM beta-mercaptoethanol), 0.25 mM dNTP mixture, 0.5 μM each primer and 1.0 U of Hot Start Taq polymerase (TaKaRa). Touchdown PCR was then carried out using the primer sequences and restriction enzymes listed in Table 1

**Table 1:**
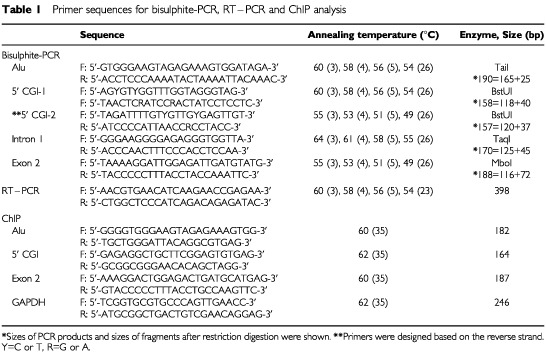
Primer sequences for bisulphite-PCR, RT–PCR and ChIP analysis

. Primers were designed based on the nucleotide sequences obtained from Genbank (AL591852). Samples (20 μl) of PCR product were digested with restriction enzymes that cleave CpG sites retained because of methylation. After ethanol precipitation, the DNA was subjected to 3% agarose gel electrophoresis and stained with ethidium bromide.

### RT–PCR

Total RNA was prepared from samples of normal stomach and colon tissue, lymphocytes and cancer cell lines, after which 5 μg samples were reverse-transcribed using Superscript II (GIBCO) to prepare first strand cDNA. Primer sequences and PCR parameters are shown in Table 1. Controls consisted of RNA treated identically but without the addition of reverse transcriptase and are labelled as RT. The integrity of the cDNA was confirmed by amplifying GAPDH as described previously (Suzuki *et al*, 2000). Samples (10 μl) of amplified product were then subjected to 2.5% agarose gel electrophoresis and stained with ethidium bromide.

### Chromatin immunoprecipitation analysis (ChIP)

ChIP was performed as described previously (Magdinier and Wolffe, 2001). Briefly, DNA was crosslinked with chromatin by incubating cells in 1% formaldehyde for 10 min at 37°C. The cells were then washed with ice-cold PBS containing protease inhibitors and resuspended in lysis buffer (1% SDS, 10 mM EDTA, 50 mM Tris-HCl, pH 8.0, and protease inhibitor). The DNA with chromatin was then fragmented into 200–1000 bp segments by sonication, after which immunoprecipitation was carried out for 16 h at 4°C using anti-acetylated histone H3 antibody (Upstate Biotechnologies, Lake Placid, NY, USA) as a probe. The resultant immune complexes were collected using protein A-agarose, after which the DNA was purified by phenol/chloroform extraction, precipitated with ethanol and resuspended in water. About 1 out of 100 of the precipitated DNA was used for PCR; 1 out of 100 of the solution before adding antibody was used as internal control for the amount of DNA. PCR was performed in solution containing 1×PCR buffer (TaKaRa), 1 μM primers, 0.25 mM dNTP mixture and 1.0 U of Hot Start Taq polymerase (TaKaRa). The primer sequences for the PCR reaction are shown in Table 1. The amplified products were subjected to agarose gel electrophoresis, and the intensity of resultant bands was calculated using a Lane and Spot Analyzer (Atto, Japan).

## RESULTS

**Figure 1:**
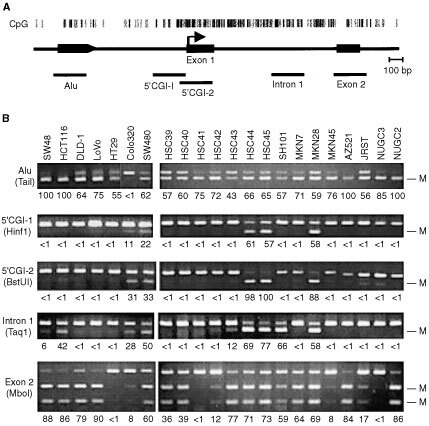
Analysis of DAPK CGI methylation. (**A**) CpG sites are shown as vertical bars; exons and Alu are shown as solid boxes. The regions analysed are shown below the line. (**B**) Representative results of a COBRA of the DAPK CGI carried out in a panel of colorectal and gastric cancer cell lines. The methylation status of the five indicated regions of the DAPK CGI was examined by bisulphite-PCR using appropriate primers. The regions and restriction enzymes (in parenthesis) used are shown on the left. The degree of methylation was calculated by densitometry and are shown as percentages below the gels; M, methylated alleles.

DAPK contains a CGI that spans approximately 2 kb in its 5′ region. By comparing the genomic sequences obtained by the human genome project (AL591852) with cDNA sequences (AU100257) obtained from a 5′-end-enriched cDNA library constructed using the oligo-capping method (Suzuki *et al*, 1997), the transcription start site of DAPK was determined to be 239 bp upstream from the boundary between exon 1 and intron 1 (Figure 1A

, arrow). To examine the methylation status of DAPK in colorectal and gastric cancers, five primer sets were designed to span the entire DAPK CGI (Figure 1A). Among the 26 cell lines analysed, 96% (25 of 26) showed methylation of Alu, situated at the 5′ edge of the CGI, while 88% (23 of 26) showed methylation of exon 2, situated at the 3′ edge (Figure 1B). By contrast, methylation of the 5′ CGI and intron 1 was detected in only 23% (6 of 26) and 35% (9 of 26) of cell lines, respectively, and all cells showing methylation of the 5′ CGI also showed methylation of intron 1 (Figure 1B).

**Figure 2:**
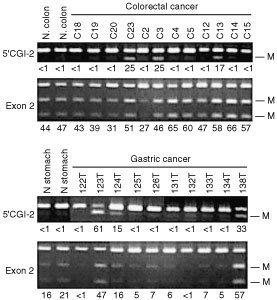
Bisulphite-PCR analysis of the DAPK CGI in primary colorectal and gastric cancers. The regions analysed are shown on the left. The degree of methylation was calculated by densitometry and are shown as percentages below the gels; M, methylated alleles; T, tumour, N, adjacent nontumourous tissue.

To examine the methylation status of DAPK in primary tumours, COBRA was performed using samples of bisulphite-treated DNA from 28 colorectal and 27 gastric cancer cases and a corresponding sample of normal gastric mucosa (Figure 2

). Methylation of exon 2 was detected in virtually every tumour analysed, whereas methylation of the 5′ CGI was detected in only 14% (4 of 28) colorectal and 15% (4 or 27) of gastric cancer cases.

**Figure 3:**
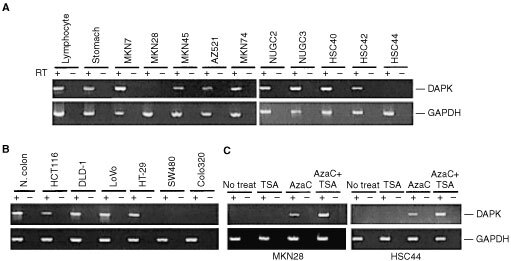
Expression of DAPK in the indicated gastric (**A**) and colorectal (**B**) cancer cell lines was analysed by RT–PCR. Controls consisted of carrying out PCR reactions without reverse transcription (RT-) and amplification of GAPDH to assess the quality of the cDNA. (**C**) Effects of methyltransferase and/or histone deacetylase inhibitor on the expression of DAPK. The indicated cell lines were treated with either 1 μM 5′-aza-dC, 300 nM TSA or both.

To assess DAPK expression, RT–PCR was performed using cDNA prepared from normal tissues and cancer cell lines (Figures 3A,B). Eight of 10 gastric and 5 of 7 colorectal cancer cell lines expressed DAPK at readily detectable levels, while four cell lines (MKN28, HSC44, Colo320 and SW480) showed no expression at all. To confirm the role of DNA methylation in the silencing of DAPK expression, two of the cell lines not expressing DAPK (MKN28 and HSC44) were treated with 5-aza-dC, a methyltransferase inhibitor, and/or TSA, a histone deacetylase inhibitor. Expression of DAPK was partially restored by treating the cells with 5-aza-dC, and addition of TSA synergistically enhanced the effect (Figure 3C

). Treatment with TSA alone had no affect on gene expression.

**Figure 4:**
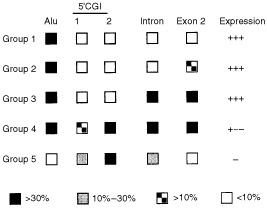
Summary of methylation levels in colorectal (CRC) and gastric cancer (GC) cell lines, which were divided into five groups based on the methylation density of each region. The percentages were calculated by densitometry and are indicted by the boxes. Group 1, CRC: HT29, GC: HSC41, MKN45, NUGC3 and NUGC4 cells; group 2, CRC: Caco2, SW48, DLD-1, LoVo, GC: HSC39, HSC40, HSC42, MKN7, MKN74, AZ521, JRST and NUGC2 cells; group 3: CRC: HCT116, GC: HSC43 and SH101; group 4: CRC: RKO, SW480, GC: MKN28, HSC44 and HSC45; group 5: CRC: Colo320.

Summary of methylation level of each region and expression of DAPK is shown in Figure 4

. We found that the cell lines could be divided into five groups based on the methylation densities determined by COBRA. Although methylation of Alu and exon 2 occurred frequently, such methylation at the edge of the CGI did not affect DAPK expression, and all cell lines belonging to groups 1, 2 and 3 expressed DAPK mRNA at readily detectable levels. On the other hand, methylation of the 5′ CGI was well correlated with loss of expression. It is also notable that, with the exception of Colo320, which was methylated at the 5′ CGI but not at Alu or exon 2, cell lines showing methylation of 5′ CGI typically showed substantial methylation of the entire CGI.

**Figure 5:**
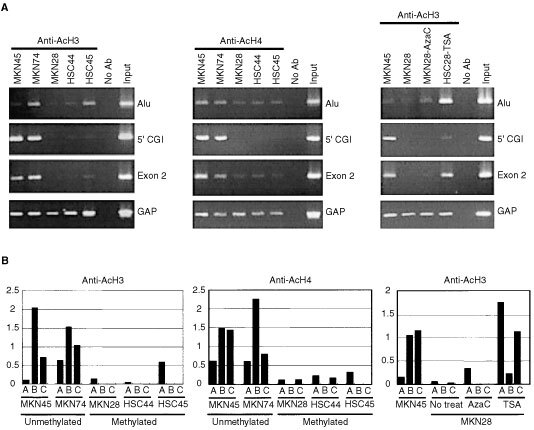
Acetylation status of histones H3 and H4 in gastric cancer cell lines. (**A**) ChIP-PCR analysis of various regions of DAPK CGI was performed using the primers indicated on the right, after which samples (10 μl) of the PCR product were subjected to electrophoresis and stained with ethidium bromide. Band intensities were normalized to those of amplified DNA fragments from the 5′ region of GAPDH. (**B**) Quantitative analysis of histone acetylation. The bars illustrate the relative acetylation levels of histones H3/GAPDH and histone H4/GAPDH calculated by densitometry. The regions analysed are shown below the column: (**A**) Alu; (**B**) 5′CGI; (**C**) exon 2.

To examine the histone acetylation status in the region around the DAPK CGI, ChIP assays were performed using antibodies against acetylated histone H3 and H4 (Figure 5A

), enabling relative levels of histone acetylation were determined for each region of the DAPK CGI (Figure 5B). Overall, acetylation of both histone H3 and H4 were found to correlate directly with gene expression and inversely with DNA methylation in the region around the transcription start site (5′ CGI). It is notable that histone acetylation at the edge of the CGI (Alu and exon 2) also correlated with gene expression, regardless of whether the DNA was methylated. Levels of histone acetylation were enhanced slightly by treatment with 5-aza-dC and were significantly restored by treatment with TSA.

## DISCUSSION

DAPK is a pro-apoptotic serine/threonine kinase whose expression is induced by interferon-γ (Deiss *et al*, 1995), and whose inactivation by DNA methylation of its promoter region is associated with various human tumours (Kissil *et al*, 1997; Katzenellenbogen *et al*, 1999; Esteller, 2000; Kim *et al*, 2001; Tang *et al*, 2000; Dong *et al*, 2001). Indeed, Kang *et al* (2001) recently reported that the DAPK gene is methylated in 30% of gastric cancers and in 30% of samples of gastric mucosa from regions adjacent to the cancers. Still, the precise relationship between DNA methylation and gene expression remains unclear. For instance, DAPK has a relatively large CGI at its 5′ end, and it was not known whether the entire CGI is methylated in cancer cells or whether regional methylation is sufficient to silence gene expression. To clarify this issue, we used a semi-quantitative methylation assay to assess the methylation status of the entire DAPK CGI in a large panel of colorectal and gastric cancer cells. Our findings indicate that dense methylation of the region around the transcription start site is closely associated with DAPK gene silencing.

Our findings also suggest that the edge of the CGI is more susceptible to methylation in cancer cells than more central regions, but this does not cause gene silencing, and the functional consequences of methylating the edge of the CGI remain unknown. One attractive hypothesis is that methylation of the edge–for example, at transposons such as Alu and B1 or at simple repetitive sequences as shown in E-cadherin and GST-P (Graff *et al*, 1997; Yates *et al*, 1999; Millar *et al*, 2000) serves as a trigger for spreading methylation into the centre of the region. In fact, Alu sequences situated about 500 bp upstream of the DAPK 5′ CGI are densely methylated in a large majority of samples, suggesting the role for repetitive elements as a trigger for 5′ CGI methylation. Methylation of exon 2, was also frequently detected in cell lines, regardless of gene expression, which is consistent with earlier findings that methylation downstream of the CGI does not affect gene silencing (Jones, 1999; Salem *et al*, 2000).

Among the six cell lines that showed methylation of the 5′ CGI, all showed methylation of intron 1. Although methylation of intron 1 is not sufficient to silence DAPK expression, it may facilitate the spread of methylation from exon 2 to the 5′ CGI. One exception to that scenario was seen in Colo320 cells, where the 5′ CGI was methylated, but Alu and exon 2 were not. In that case, methylation may be redistributed in some way, rather than simply spread. Further investigation will be necessary to determine how methylation spreads into the centre of the DAPK CGI, which is normally strongly protected from methylation.

Recent studies have shown that histone deacetylation also plays a key role in methylation-induced gene silencing (Bird and Wolffe, 1999; Cameron *et al*, 1999; Nguyen *et al*, 2001). Consistent with that idea, our ChIP results showed DNA methylation and histone acetylation to be inversely related in the region around the transcription start site. Restoration of histone acetylation by treatment with TSA, a histone deacetylase inhibitor, did not restore gene expression, however; furthermore, DNA methylation silenced gene expression even in the presence of histone acetylation. Cell lines showing dense methylation of the 5′ CGI tended to show low levels of histone acetylation at both the 5′ CGI and at edge of the CGI; thus function of the whole 5′ region of the gene appears to be linked to histone deacetylation, which is followed by chromatin condensation in these cell lines. In contrast, cell lines not methylated at the 5′ CGI showed acetylated histone at the edge of island, regardless of whether the DNA was methylated or not, which suggests the association between DNA methylation and histone acetylation is regulated differently in the 5′ CGI and at the edge of the CGI.

As the level of DAPK expression is unaffected by exposure to a DNA-damaging agent in unmethylated cells (data not shown), activation of DAPK is believed to be controlled at a post-transcription level. In fact, Shohat *et al* (2001) demonstrated that DAPK activity is controlled by phosphorylation of Ser308 within the CaM regulatory domain. The role of DAPK in the tumorigenesis of gastrointestinal cancer remains unknown, but in malignant lymphoma, loss of DAPK reduces responsiveness to interferon-γ (Katzenellenbogen *et al*, 1999). In addition, DAPK was recently shown to be involved in activation of a p53-dependent apoptotic pathway, and its loss appears to result in inactivation of p53 in tumours (Raveh *et al*, 2001). Conversely, restoration of DAPK to physiological levels in a highly metastatic mouse lung carcinoma model, in which DAPK expression was otherwise silenced, strongly reduced the metastatic capacity of the disease (Inbal *et al*, 1997). It therefore seems likely that loss of DAPK confers a selective advantage to cancer cells and may play a causative role in the metastasis of gastrointestinal cancer. Thus, DAPK may be a useful molecular marker suggesting the prognosis of gastrointestinal cancers. Because the number of cases we analysed in this study was too small to find correlation between DAPK methylation and metastasis, it is necessary to determine such correlation using large number of cases.

In summary, we have shown that regional DNA methylation and histone deacetylation plays a key role in silencing DAPK gene expression in colorectal and gastric cancers. Inhibition of DNA methylation and histone deacetylation acted synergistically to induce gene expression, suggesting that DAPK may be an effective molecular target for the treatment of a subset of colorectal and gastric cancers through activation of apoptosis using methyltransferase and histone deacetylase inhibitors.
